# Response of the authors regarding article: “Association between electrocardiographic features and mortality in COVID‐19 patients”

**DOI:** 10.1111/anec.12886

**Published:** 2021-09-02

**Authors:** T. David Gbadebo, Daniel Antwi‐Amoabeng, Bryce D. Beutler, Mark B. Ulanja

**Affiliations:** ^1^ Emory Decatur Hospital Cardiology Section Decatur GA USA; ^2^ Department of Internal Medicine University of Nevada Reno School of Medicine Reno NV USA

Dear Editor,

The authors of the recently published article “Association between electrocardiographic features and mortality in COVID‐19” (Antwi‐Amoabeng et al., 2021) find it necessary to respond to the commentary submitted by Mahmoudi et al (Mahmoudi et al., 2021). We could not appreciate any challenges to any of our findings, nor did we garner any editorial highlights for clarification or illumination. The greatest part of their submission was devoted to a lengthy discussion on methodology and approaches for measuring QT interval or indices of ventricular repolarization. Baseline QT interval was measured among our patient population due to some background exposure to QT‐prolonging drugs. However, we did not find nor report any disparate clinical ventricular arrhythmias or sudden cardiac death in our study. We reported disparate findings of sinus tachycardia and atrial fibrillation that appear to portend an increased risk of death. Different parameters of risk assessment for ventricular arrhythmias have been proposed in experimental animal and clinical models (Gbadebo et al., 2002), but these are not germane to our referenced article. We acknowledge that some aspects of our findings and reports are limited and can be better refined. Another recent publication has incorporated data from our article in a meta‐analysis examining the impact of LBBB on COVID‐19 disease (Zuin et al., 2021). We welcome thoughtful, worthy, and pertinent commentaries that can further educate, illuminate, and advance our understanding of risk predictors in patients with COVID‐19.

Sincerely,

T. David Gbadebo, MD, FACC, FHRS

Daniel Antwi‐Amoabeng, MD, MSc

Bryce D. Beutler, MD

Mark B. Ulanja, MD, MPH
